# Chemotherapy-induced exosomal circBACH1 promotes breast cancer resistance and stemness via miR-217/G3BP2 signaling pathway

**DOI:** 10.1186/s13058-023-01672-x

**Published:** 2023-07-17

**Authors:** Wenjie Xia, Wuzhen Chen, Chao Ni, Xuli Meng, Jun Wu, Qiong Yang, Hongchao Tang, Hongjun Yuan, Shan Fang

**Affiliations:** 1 General Surgery, Cancer Center, Department of Breast Surgery, Zhejiang Provincial People’s Hospital, Affiliated People’s Hospital, Hangzhou Medical College, 158 Shangtang Road, Hangzhou, 310014 Zhejiang China; 2 Department of Breast Surgery (Surgical Oncology), Second Affiliated Hospital, Zhejiang University School of Medicine, Hangzhou, China; 3 Center for Rehabilitation Medicine, Rehabilitation & Sports Medicine Research Institute of Zhejiang Province, Department of Rehabilitation Medicine, Zhejiang Provincial People’s Hospital, Affiliated People’s Hospital, Hangzhou Medical College, 158 Shangtang Road, Hangzhou, 310014 Zhejiang China

**Keywords:** Paclitaxel, Chemoresistance, Exosomes, circBACH1, Breast cancer

## Abstract

**Background:**

Chemoresistance involves metastasis and aggressiveness of breast cancer (BC). Chemotherapy-elicited exosomes have been reported to be associated with drug resistance and pro-metastatic capacity of BC cells. Non-coding RNAs (ncRNAs) are enriched in exosomes, which participated in generation, progression, and resistance of BC. However, the mechanism underlying the chemoresistance and metastasis in BC cells mediated by the BC-derived exosomal ncRNAs remained to be elucidated.

**Methods:**

The effects of PTX-induced exosomal circBACH1 on BC cell function were assessed using RNA Binding Protein Immunoprecipitation (RIP), dual luciferase reporter gene, tube formation, CCK-8, and Western Blot assays. The circBACH1 and miR-217 expression levels were detected using quantitative real-time PCR (RT-qPCR) and Immunohistochemistry (IHC) assays in BC tissues and precancerous tissues of BC patients.

**Results:**

CircBACH1 expression was increased in paclitaxel-treated BC-derived exosomes (PTX-EXO) and BC tissue. PTX-EXO was shown to promote PTX-resistance and angiogenesis through upregulation circBACH1. Downregulation of circBACH1 improved PTX-sensitiveness by suppressing the cell viability, stemness, migration, and angiogenesis of BC cells. Moreover, we found that miR-217 interacted with circBACH1 and targeted GTPase-activating SH3 domain-binding protein 2 (G3BP2) in BC cells. CircBACH1 combined miR-217 cotransfection suppressed the expression of G3BP2 proteins compared with circBACH1 treatment in MCF-7 cells. In addition, downregulation of G3BP2 suppressed BC cell migration.

**Conclusions:**

These results demonstrated that PTX-induced exosomal circBACH1 promoted stemness and migration of BC cells by sponging miR-217 to upregulate the expression of G3BP2, which provided a new therapeutic target for PTX-resistance and progression of BC via circBACH1/miR-217/G3BP2 axis.

**Supplementary Information:**

The online version contains supplementary material available at 10.1186/s13058-023-01672-x.

## Background

Breast cancer (BC) is one of the most rapidly increased causes of cancer mortality worldwide [1]. The incidence (11.7%) and mortality (6.9%) of BC ranked first in female among all cancers globally in 2020 [1–3]. The treatment strategies for BC vary from local treatment including surgery and radiation to systemic treatment including hormonal therapy, endocrine therapy, chemotherapy, biological therapy, or immunotherapy [4]. Customized treatments are recommended for BC patients nowadays according to tumor subtype, cancer stage, genetic factors, patient preferences, and the efficiency of neoadjuvant therapy [5, 6]. Neoadjuvant therapy was chemical agents or endocrine drugs given before local treatment for BC to optimize subsequent resection or radiotherapy regimen [7]. Neoadjuvant chemotherapy has developed as a standard of care for locally advanced BC or BC patients with poor pathological features to shrink tumors, prevent metastasis, or assess chemosensitivity [8–11]. Previous studies reported that platinum-based neoadjuvant chemotherapy combined with carboplatin treatment effectively improved pathological complete response and survival in triple negative breast cancer [12, 13]. However, drug resistance to neoadjuvant chemotherapy may lead to treatment failure and promote the progression of BC [14]. A latest study reported that chemotherapy could induce drug resistance and stemness of BC cells by activation of the EZH2/STAT3 pathway to secret miR-378a-3p and miR-378d containing exosomes [15]. Therefore, it’s urgent to explore a novel method to resolve the chemoresistance and metastasis of BC in clinic.

Exosomes are extracellular micro-vesicles containing various proteins and nucleic acids, which play roles in transporting information and substance among cells [16, 17]. Previous studies have shown that chemotherapy-elicited exosomes are associated with the drug resistance and pro-metastatic capacity of BC cells [18, 19]. Enriched-ANXA6 in exosomes derived from taxanes and anthracyclines treated tumors led to the chemoresistance and pro-metastatic effects in BC [18, 20, 21]. CircRNA, a non-coding circular RNA enriched in exosomes, which involves in the generation, progression, and resistance of BC [22]. A previous study showed that downregulation of circWAC increased PTX-sensitivity in triple-negative BC by increasing miR-142 to inhibit WWP1 expression and suppress the PI3K/AKT pathway [23]. CircBACH1 was reported to promote hepatocellular carcinoma progression by inhibiting the translation of p27 via HuR [24]. Another study showed that circBACH1 accelerated colorectal cancer progression by reducing let-7a-5p to increase CREB5 expression [25]. However, the effects of exosome-derived circBACH1 on the progression of BC and the potential mechanism of exosomal circBACH1 regulating the chemoresistance and metastasis of BC remain to be elucidated.

Previous studies reported that the overexpression of G3BP stress granule assembly factor 2 (G3BP2) promoted the proliferation and metastasis in BC [26, 27]. A latest study showed that G3BP2 maintained long-term proliferative properties of BC cells by regulating the expression of SART3, Nanog, and Oct-4 [26]. On the other hand, inhibition of G3BP2 suppressed bladder cancer progression by upregulating circFNDC3B to reduce miR-1178-3p and inhibit the downstream SRC/FAK signaling pathway [28]. In this study, we clarified that downregulation of circBACH1 inhibited the PTX resistance and stemness of BC cells. Our study showed that PTX-induced exosomal circBACH1 mediated the PTX resistance and stemness of BC cells by sponging mirRNA-217 to increase G3BP2 expression. These results provided a novel therapeutic strategy for the chemoresistance and metastasis of BC.

## Methods

### Cells culture

The human BC cell lines MDA-MB-231, MDA-MB-468, MCF-7 were obtained from the Cell Bank of the Chinese Academy of Sciences (Shanghai, China). The non-cancerous mammary epithelial cell line MCF-10A the human umbilical vein endothelial cells (HUVECs) were purchased from ATCC (Manassas, VA, USA). The MCF-7, HUVECs, MDA-MB-468 and MDA-MB-231 cells were cultured in RPMI 1640 (Gibco) containing 10% FBS at 37 °C under a humidified chamber supplemented with 5% CO_2_. The MCF-10A cells were cultured in DMEM/F12 (invitrogen, Carlsbad, CA, USA) containing 10% FBS under a humidified atmosphere with 5% CO_2_ at 37 °C.

### Clinical specimen

A total of 9 BC patients were collected from 2021 to 2022 in Zhejiang Provincial People’s Hospital. The Blood of BC patients were collected before and after PTX treatment. The BC tissues and precancerous tissues were collected from the BC patients, then stored at − 80 °C for further analysis. The written informed consent forms were signed by all the BC patients. All the clinical experiments were approved by the Ethics Committee of Zhejiang Provincial People’s Hospital (No. 2022268).

### Exosomes purification and identification

Cells were treated with 100 nmol/l PTX, or PBS as control for 48 h. Then the supernatants of the medium were collected, which were centrifuged at 500*g* for 5 min, 2000*g* for 15 min, and 10,000*g* for 20 min at 4 °C to remove cells, debris, and large vesicles. Exosomes were obtained by ultracentrifugation of the supernatants at 110,000×*g* for 70 min. Exosomes were resuspended in PBS for further analysis. which were validated by transmission electron microscopy and Western blotting. ExoQuick Exosome Precipitation Solution (SBI System Biosciences, Mountain View, CA, USA) was used to isolate exosomes from plasma.

### CircRNA microarray

Total RNA extracted from exosomes derived from PTX-treated normal mammary epithelial cells (n = 4) and exosomes derived from PTX-treated MCF-7 cells (n = 6) RNA digestion was performed using RNase R (Epicentre, Madison, WI, USA) to remove linear RNA. According to the manufacturer’s protocol of the cRNA Amplification and Labeling Kit (KangChen, Shanghai, China), random primers were used to amplify the enriched circRNA and were subsequently transcribed into fluorescent circRNA. The amplified circRNA was purified using the RNeasy Mini kit (Qiagen, Düsseldorf, Germany). The labeled RNA was hybridized to the human circRNA Array (KangChen, Shanghai, China). The circRNA array was designed to have four identical arrays per slide, and each array contained probes to interrogate approximately 13,524 human circRNAs. Each circRNA was detected simultaneously by one long probe and one short probe.

### RNA extraction and quantitative real-time PCR (RT-qPCR)

The total RNA extraction was isolated from BC tissues and BC cells using the RNeasy Mini Kit (Qiagen, USA). Then the RNAs were converted into cDNA with the reverse Transcription Kit (Qiagen). The miScript SYBR Green PCR Kit (Qiagen) was used for amplification of cDNA and the RT-qPCR was performed on ABI PRISM 7500 real-time PCR System. The amplification program was set as follows: 50 °C for 2 min, 95 °C for 10 min followed by 50 cycles of 95 °C for 15 s, 72 °C for 30 s, 72 °C for 5 min. The forward primers of circBACH1: 5′-ACTGTGGATCAGCTCCCTTTG-3′, reverse primers of circBACH1: 5′-CACAGCCAATGTGTGAACCT-3′. The forward primers of miR-217: 5′-TACT GCATCAGGAACTGATTGGA-3′, reverse primers of miR-217: 5′-TCCAATCAGTTCCTGATG-3′. The forward primers of G3BP2: 5′-CCGGCAGAACCTGTTTCTCT-3′, reverse primers of G3BP2: 5′-GACCTCGATCATTGCGCCTA-3′. The relative RNA expression was calculated by the standard 2^−ΔΔCt^ method and the experiments were repeated three times.

### Transmission electron microscopy

The isolated exosomes were fixed using 4% paraformaldehyde. Then the fixed exosomes were adsorbed onto a copper grids. The copper grids with adsorbed exosomes were stained with 2% uranyl acetate for 30 s. At last, the images were captured by the transmission electron microscopy (TEM, JEOL,USA).

### Western blot analysis

The lysis buffer (100 mM Tris–HCl, 2% SDS, 1 mM Mercaptoethanol, 25% Glycerol) was used to extract the proteins. The BCA Protein Assay Kit (Beyotime, China) was applied to quantify the proteins. The proteins were separated by 10% polyacrylamide gel electrophoresis (SDS-PAGE), then they were transferred to nitrocellulose (NC) membrane. The NC membranes were blocked with 5% BSA for 1 h at room temperature. After washed with PBS, they were incubated with the primary antibody against CD81 (1:1000, Abcam, Cambridge, UK), CD63 (1:1000, Abcam, Cambridge, UK), TSG101 (1:1000, Abcam, Cambridge, UK), CD44 (1:1000, Abcam, Cambridge, UK), CD133 (1:1000, Abcam, Cambridge, UK), Oct-4 (1:1000, Abcam, Cambridge, UK), G3BP2 (1:1000, Cell Signaling Technology, Danvers, MA, USA), β-actin (1:1000, Cell Signaling Technology, Danvers, MA, USA) at 4 °C overnight. Next, the secondary antibody (1:7500, Abcam, Cambridge, UK) was incubated with the NC membranes at room temperature for 2 h. Finally, the chemiluminescence detection system was performed to visualize the protein bands, which was analyzed by the Image J software (NIH).

### Subcellular fractionation

MCF-7 cells were collected and used for nuclear and cytoplasmic RNA detection. For nuclear and cytoplasmic RNA separation, the cells were collected and extracted using a PARIS kit (Life Technologies, Carlsbad, CA, USA).

### Fluorescence in situ hybridization (FISH) assay

The circBACH1 probes labeled by Cy3 were designed and synthesized by GenePharma (Shanghai, China). The circBACH1 probe was: 5′-GTCACCAGCTTCTCAATTTTT-3′. The probes were incubated in water at 75 °C for 5 min and immediately at 0 °C for 10 min for annealing. The cells were fixed with solution containing 4% paraformaldehyde and 0.5% Triton X-100 for 20 min. After dehydration with 70%, 95% and 100% ethanol, the cells were incubated with the annealed probes on the slides at 37 °C overnight for hybridization. The slides were incubated in washing buffer at 48 °C for 20 min after washed with hybridization buffer. The 4,6- diamidino- 2- phenylindole (DAPI, Beyotime) was added to stain the cyteblasts before sealed with Nail Polish. The results were observed for four fields using a fluorescence microscope (Leica, Wetzlar, Germany) and analyzed by ImageJ software (NIH, Bethesda, MD, USA).

### Northern blot

Northern blot was performed as previously described (29). Briefly, 10 µl RNA was electrophoresed and transferred onto a positively charged nylon membrane (GE Healthcare, Shanghai, China). Hybridization was performed overnight with biotin-labeled circBACH1 probe. GAPDH was used as control. The membranes were incubated with HRP-linked streptavidin for 15 min, then incubated with chemiluminescent substrate and the blots were detected using the chemiluminescent detection system.

### Mammosphere formation assay

After treatment with PTX-EXO and/or si-circBACH1 for 24 h, cells were seeded at a density of 5 × 10^4^, and cultured in DMEM/F12 supplemented with B27 (Invitrogen), 20 ng/ml EGF (Sigma-Aldrich), 10 ng/ml bFGF (Sigma-Aldrich), and 20 ng/ml insulin-like growth factor I (Invitrogen) for 7 days. Mammosphere was calculated by larger than 50 μm in diameter under a microscope (Olympus, Tokyo, Japan).

### Flow cytometry analysis

PTX-EXO and/or si-circBACH1 treated cells in the presence of PTX were dual-labeled with a APC-conjugated anti-CD44 antibody and an FITC-conjugated anti-CD24 antibody (BD Pharmingen, San Diego). 1 × 10^5^ cells were labeled and incubated with FITC-conjugated anti-CD24 and APC-conjugated anti-CD44 antibody at 4 °C in the dark for 1 h. The cells were washed and then analyzed by flow cytometer (BD FACS Aria II).

### Cell viability

The BC cells were harvested to detect the cell viability by CCK-8 (Dojindo, Kumamoto, Japan) according to the manufacturer’s instructions. Briefly, 100 μl Cell suspension containing 5 × 10^3^ cells were seeded onto 96-well culture plates with corresponding treatment. After incubation with PTX (100 nmol/l), PBS-EXO, PTX-EXO, si-circBACH1 treatment for 0 h, 24 h, 48 h, 72 h, 96 h, 10 μl CCK-8 solution was added and incubated for 2 h at 37 °C respectively. Then, the absorbance at 450 nm was determined with a microplate reader (ELX800, BioTeK, USA).

### MiRNA, circRNA construction and cell transfection

The miR-217 mimics, inhibitors, si-circBACH1, and the negative control (NC) were constructed and synthesized by Sangon Biotech (Shanghai, China). The circRNA overexpression vector (#GS0108) was obtained from Geneseed (Guangzhou, China). The BC cells were transfected with the circBACH1 vectors, miR-217 inhibitors or si-circBACH1 using the Lipofectamine 2000 (Invitrogen, Carlsbad, CA, USA) with the manufacturer’s protocol.

### Transwell assay

The Matrigel-coated transwell cell culture chambers (BD Matrigel Invasion Chamber, BD Biosciences, USA) was applied to determine the migration capability of the BC cells with corresponding treatment. About 1 × 10^4^ BC cells were suspended in 200 μl medium without serum and seeded into the upper chambers of each transwell (8 μm pore size) coated with Matrigel (BD Biosciences, USA). Medium containing 10% FBS was added to the bottom chamber. After incubation with PTX, PBS-EXO, PTX-EXO, si-circBACH1 treatment for 24 h, cells in the upper chamber were removed with cotton swabs and the cells on the bottom chamber were fixed by 4% formaldehyde for 15 min, stained for 20 min using 0.1% crystal violet. An inverted light microscope was applied to count the migration cells (Olympus, Tokyo, Japan).

### In vitro tube formation assay

HUVECs were cultured in DMEM without serum for 24 h, then transferred into 24-well plates pre-coated with Matrigel (Invitrogen) and stimulated for 12 h with MCF-7 medium post PTX, PBS-EXO, PTX-EXO or si-circBACH1 treatment. The relative length of tubes in HUVECs were measured by Image J (NIH).

### Dual luciferase assay

The wild type (WT) or the mutant type (Mut) of circBACH1 and G3BP2 gene fragments were respectively cloned into psiCHECK-2 vector (Promega, Madison, WI). For circBACH1-miR-217 luciferase assay, 0, 1, 5, 10 nm of miR-217 was co-transfected with WT or Mut circBACH1 luciferase reporter into 4 × 10^5^ MCF-7 cells, respectively. For G3BP2-miR-217 luciferase assay, 0, 1, 5, 10 nm of miR-217 was cotransfected with WT or Mut G3BP2 luciferase reporter into MCF-7 cells, respectively. After incubation for 48 h, dual Luciferase Reporter Assay System (Promega) was applied to determine the luciferase activity of the MCF-7 cells.

### RNA binding protein immunoprecipitation (RIP) assay

MCF-7 cell lysates from different groups were incubated with RIP buffer containing magnetic beads conjugated with anti-human argonaute 2 (Ago2) or IgG antibodies (Millipore, Billerica, MA, USA) overnight under 4 °C. Then streptavidin-coated magnetic beads were added for incubation for 2 h. The isolated and purified RNAs was subjected to qRT-PCR measurement. The immunoprecipitated RNAs were then extracted and detected by qRT-PCR to confirm the enrichment of binding targets and the products were then subjected to agrose gel electrophoresis. The primers used for detecting circBACH1 or miR-217 were listed in RT-qPCR.

### Immunohistochemistry (IHC)

Paraffin-embedded slides of BC tissue were dewaxed in xylol for 20 min and rinsed with 100% ethanol for the staining process. Each slide was incubated with G3BP2 (1:200, Cell Signaling Technology, Danvers, MA, USA) for 24 h at 4 °C. Immunostaining was visualized with the DAB staining system (Beyotime). These slices were Images were visualized using a microscope system (Leica, Wetzlar, Germany).

### Statistics

All experiments were repeated for 3 times. Statistics were presented as mean ± SD. Student’s t-test was used to assess the statistical significance for comparisons between two groups. One way ANOVA was used for the comparison of multiple groups (> 2). GraphPad Prism (v9.0, GraphPad Software Inc., San Diego, CA, USA) was applied for statistical analyses. P < 0.05 was considered as a statistically significant difference.

## Results

### CircBACH1 was highly expressed in the exosomes derived from PTX-treated BC cells

**Fig. 1:**
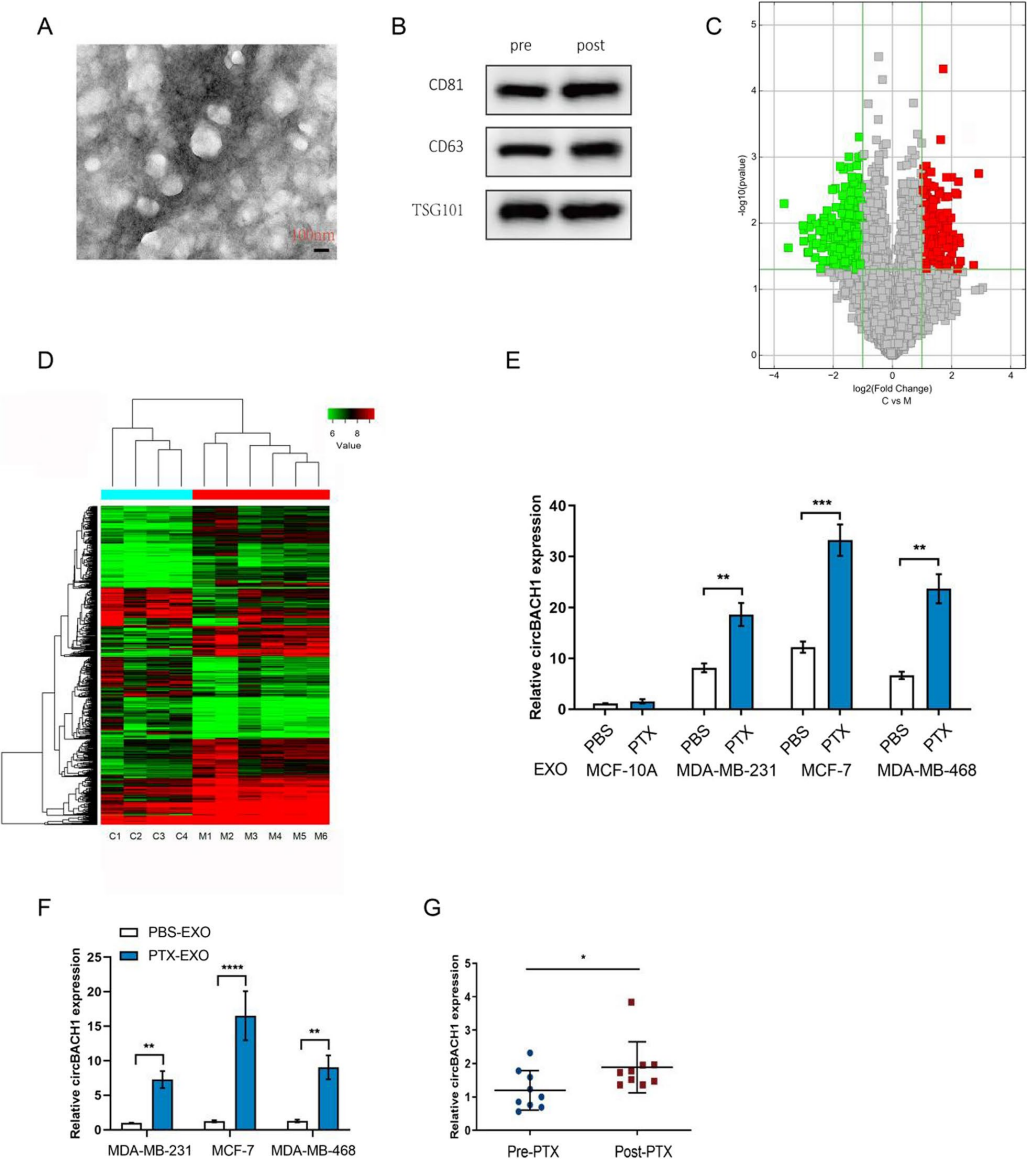
CircBACH1 was highly expressed in the exosomes derived from PTX-treated BC cells. **A** The exosomes were identified by transmission electron microscopy. **B** The surface markers of exosomes were detected by western blotting pre- or post- PTX treatment. **C** Volcano plot of the differentially expressed circRNAs. Red represents up-regulated circRNAs, and green represents down-regulated circRNAs. The vertical lines demark the fold change values, which correspond to 2.0-fold up and down. The horizontal line marks a *P* value of 0.05; **D** A heat map of cluster analysis demonstrating the different circRNA expression profiles between four gorups of exosomes derived from PTX-treated normal mammary epithelial (MCF-10A) cells (C1, C2, C3, and C4) and six gorups of exosomes derived from PTX-treated MCF-7 cells (M1, M2, M3, M4, M5 and M6). The intensity is reflected in the color scale that runs from green (low intensity) to red (high intensity). **E** CircBACH1 expression was measured by RT-PCR in the exosomes derived from human normal mammary epithelial cells (MCF-10A) and BC cells (MCF-7 and MDA-MB-231) after PBS or PTX treatment. **F** The expression of circBACH1 in MCF-7 and MDA-MB-231 was examined after incubation with PBS-EXO (the exosomes of BC cells after PBS treatment) or PTX-EXO (the exosomes of BC cells after PTX treatment). **G** CircBACH1 expression was examined in the plasma exosomes of BC patients pre- or post- PTX treatment. *P < 0.05, **P < 0.01, ***P < 0.001, ****P < 0.0001

To identify the exosomes derived from BC cells, the morphology and the surface markers of the exosomes were measured by TEM and western blotting, respectively. The representative micrograph showed that the size of exosomes is nearly 50-150 nm diameter (Fig. 1A). Moreover, the exosome markers, including CD81, CD63, and TSG101, were positively expressed pre- or post-PTX treatment (Fig. 1B). CircRNA profiles were characterized by circRNA microarray analysis between four gorups of exosomes derived from PTX-treated normal mammary epithelial (MCF-10A) cells (C1, C2, C3, and C4) and six gorups of exosomes derived from PTX-treated MCF-7 cells (M1, M2, M3, M4, M5 and M6). In total, 13,524 circRNAs were detected, and 10,411 circRNAs were identified as being dysregulated in BC cells. Among them, 5,015 were up-regulated and 5,396 were down-regulated in M group compared with C group. A volcano plot was drawn using the two factors of P value and FC value for the significantly differently expressed circRNAs obtained from the above analysis (Fig. 1C). The hierarchical clustering analysis of the differentially expressed circRNAs clearly differentiated M group from C group (Fig. 1D). As a result, circBACH1 was upregulated in the exosomes derived from PTX-treated BC cells.

**Fig. 2:**
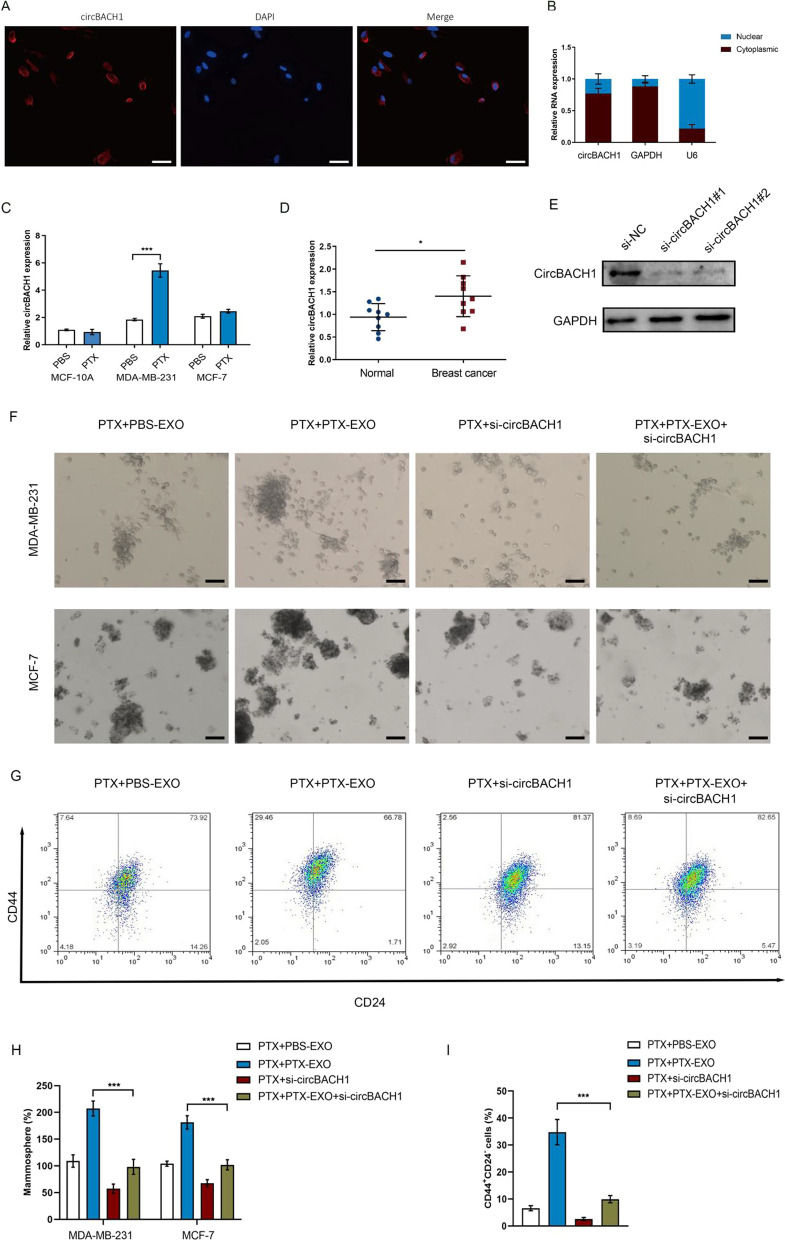
CircBACH1 was mainly located in the cytoplasm of BC cells. **A** The location of circBACH1 in MCF-7 was determined by Fluorescence in Situ Hybridization (FISH) assay, scale bar: 50 µm. **B** The expression of circBACH1 was measured in the nucleus and cytoplasm of MCF-7. **C** CircBACH1 expression was measured by RT-PCR in human normal mammary epithelial cells (MCF-10A) and BC cells (MCF-7 and MDA-MB-231) after PBS or PTX treatment. **D** CircBACH1 expression was measured in tissue of BC patients, using normal tissue as control. **E** Northern blotting of circBACH1 expression by transfection with si-circBACH1. **F**, **H** Mammosphere formation (%) was determined in MDA-MB-231 and MCF-7 cells treated with PTX, PBS-EXO or PTX-EXO, or si-circBACH1, scale bar: 50 µm. **G** Analysis of CD44 and CD24 expression in MCF-7 cells treated with PTX, PBS-EXO or PTX-EXO, or si-circBACH1 by flow cytometry. **I** The proportion of CD44^+^ CD24^−^ cells were quantified in PTX, PBS-EXO or PTX-EXO, or si-circBACH1 treated cells. *P < 0.05, ***P < 0.001

CircBACH1 was reported to promoted tumor progress in various cancer types [25, 29–31]. The expression of circBACH1 was significantly increased in the exosomes derived from PTX treated-BC cells (PTX-EXO) compared with control (Fig. 1E). To explore whether exosomes could deliver circBACH1 to the BC cells, we measureded the circBACH1 expression in MDA-MB-231 and MCF-7 post PTX-EXO treatment. The circBACH1 expression was significantly increased in the PTX-EXO-treated MCF-7, MDA-MB-231 and MDA-MB-468 cells compared with control (Fig. 1F). Interestingly, we also found that the expression of circBACH1 was significantly increased in the exosomes derived from PTX-treated BC patients compared with pre-PTX treatment (Fig. 1G). FISH assay showed that circBACH1 was mainly located in the cytoplasm of MCF-7 cells (Fig. 2A and B). Compared with control, the relative circBACH1 expression was significantly increased in the PTX-treated MDA-MB-231 cell lysates, while it was not significantly changed in the PTX-treated MCF-7 cell lysates (Fig. 2C). Consistently, the circBACH1 levels were apparently increased in the BC tissues compared with the adjacent tissues (Fig. 2D). We knocked down circBACH1 by transfection with si-circBACH1. The efficiency of si-circBACH1 transfection was characterized by northern blot (Fig. 2E). The si-circBACH1 #1 was used in the further investigation. To determine the effect of circBACH1 on the function of BC cells, the MCF-7 and MDA-MB-231 cells were treated with PTX and PTX-EXO or si-circBACH1. We observed PTX-EXO enhanced mammosphere formation in PTX treated MCF-7 and MDA-MB-231 cells, whereas loss of circBACH1 significantly attenuated mammosphere formation in PTX-EXO treated MCF-7 and MDA-MB-231 cells in the presence of PTX (Fig. 2F and H). In addition, we found that the proportion of CD44^+^CD24^−^ cells were enhanced in PTX + PTX-EXO group, whereas downregulation of circBACH1 significantly decreased the proportion of CD44^+^CD24^−^ cells in PTX-EXO treated MCF-7 cells in the presence of PTX (Fig. 2G and I).

### Downregulation of circBACH1 reversed PTX-EXO-induced cell viability and stemness

**Fig. 3:**
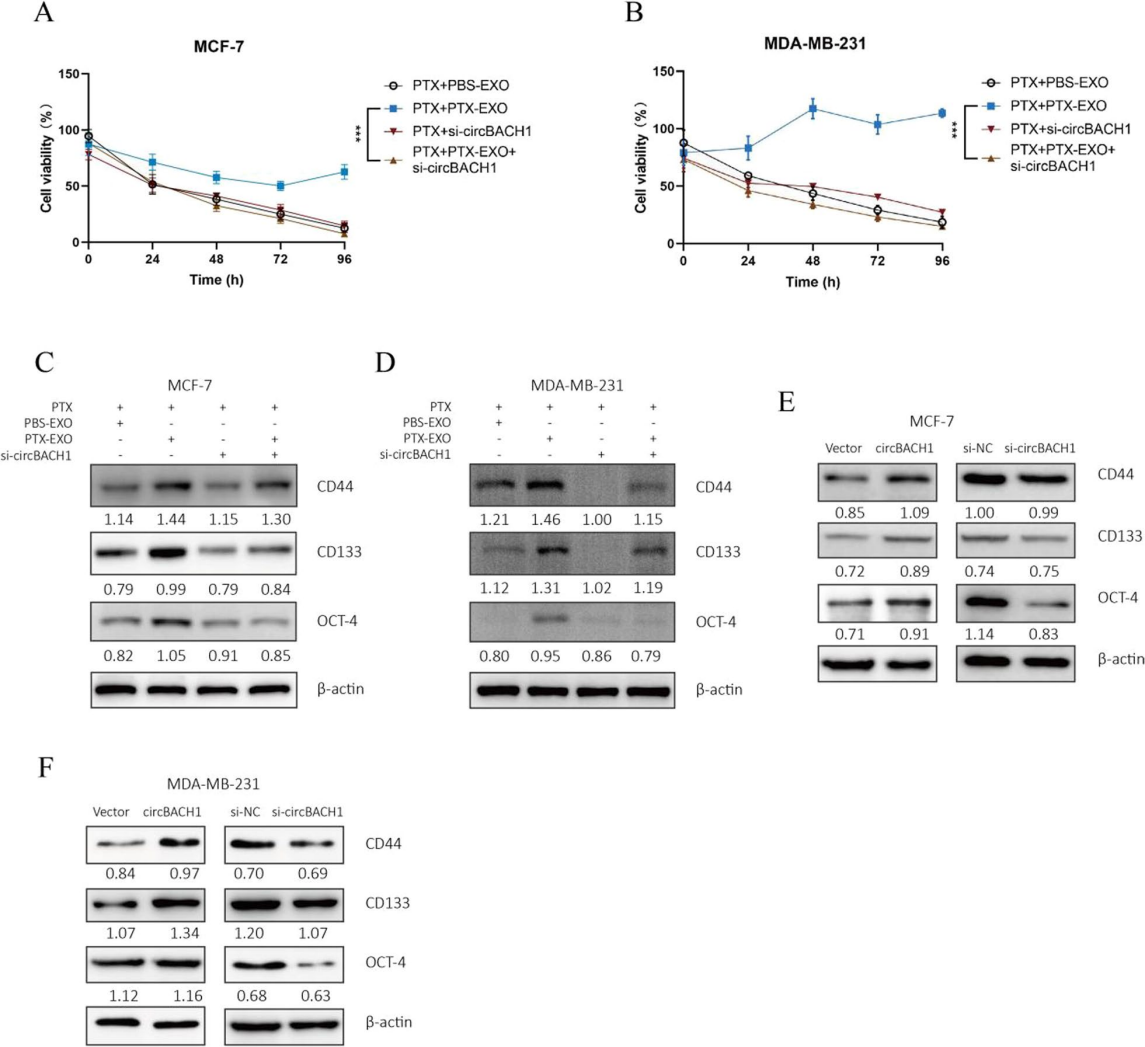
Downregulation of circBACH1 reversed the PTX-EXO-induced PTX-resistance of BC cells. **A**, **B** Cell viability were tested by CCK8 in MCF-7 (**A**) and MDA-MB-231 (**B**) treated with PTX, accompanied exosomes separated from BC cell lines with PBS treatment (PBS-EXO) or with PTX treatment (PTX-EXO), or circBACH1 inhibitor (si-circBACH1). **C**, **D** Stemness markers (CD44, CD133, Oct) of BC were measured by western blotting in MCF-7 (**C**) and MDA-MB-231 (**D**) treated with PTX, PBS-EXO or PTX-EXO, or si-circBACH1, and β-actin was set as control. **E**, **F** The expression of stemness markers in MCF-7 (**E**) and MDA-MB-231 (**F**) after transfected with vectors, circBACH1 containing vectors, si-NC, or si-circBACH1 were detected by western blotting, vetors or si-NC transfection was set as control. ***P < 0.001

To determine whether PTX-EXO mediated the cell viability and stemness in the BC cells, the MCF-7 and MDA-MB-231 cells were treated with PTX and PBS-EXO or PTX-EXO. Then we analyzed the cell viability and stemness of the BC cells after the corresponding treatment to confirm the PTX resistance of the BC cells. The cell viability of MCF-7 and MDA-MB-231 cells was markedly increased in the PTX + PTX-EXO group compared with the PTX + PBS-EXO group (Fig. 3A and B). Moreover, we compared the IC_50_ of PTX between breast cancer cells and PTX-EXO treated breast cancer cells. We found that the IC_50_ for PTX in MDA-MB-231 and MCF-7 cells was nearly 10 nM and 5 nM, respectively, whereas the IC_50_ for PTX was not achieved in PTX-EXO treated MDA-MB-231 or MCF-7 cells (Additional file 1: Fig. S1A and B). The stemness markers such as CD44, CD133, and Oct were increased in the PTX + PTX-EXO group in comparison to the PTX + PBS-EXO group in MCF-7 and MDA-MB-231 cells (Fig. 3C and D). To explore the effect of exosomal circBACH1 on PTX-resistance, circBACH1 was downregulated by si-circBACH1 in BC cells. Compared with the PTX + PTX-EXO group, the cell viability was significantly decreased in the PTX + PTX-EXO + si-circBACH1 group in MDA-MB-231 and MCF-7 cells (Fig. 3A and B). Interestingly, the expression levels of CD44, CD133, and Oct were reduced in the PTX + PTX-EXO + si-circBACH1 group in comparison to the PTX + PTX-EXO group in MDA-MB-231 and MCF-7 cells (Fig. 3C and D). Consistently, the expression of CD44, CD133, Oct were obviously increased by transfected with circBACH1 vectors compared with control, whereas decreased by transfected with si-circBACH1 compared with si-NC (Fig. 3E and F). The overexpression of circBACH1 was determined by northern blot (Additional file 1: Fig. S1C). Collectively, the results revealed that PTX-EXO promoted the PTX-resistance of BC cells through enhancing cell viability and stemness, which was reversed by the downregulation of circBACH1.

### Downregulation of circBACH1 reversed PTX-EXO-induced migration and angiogenesis

**Fig. 4:**
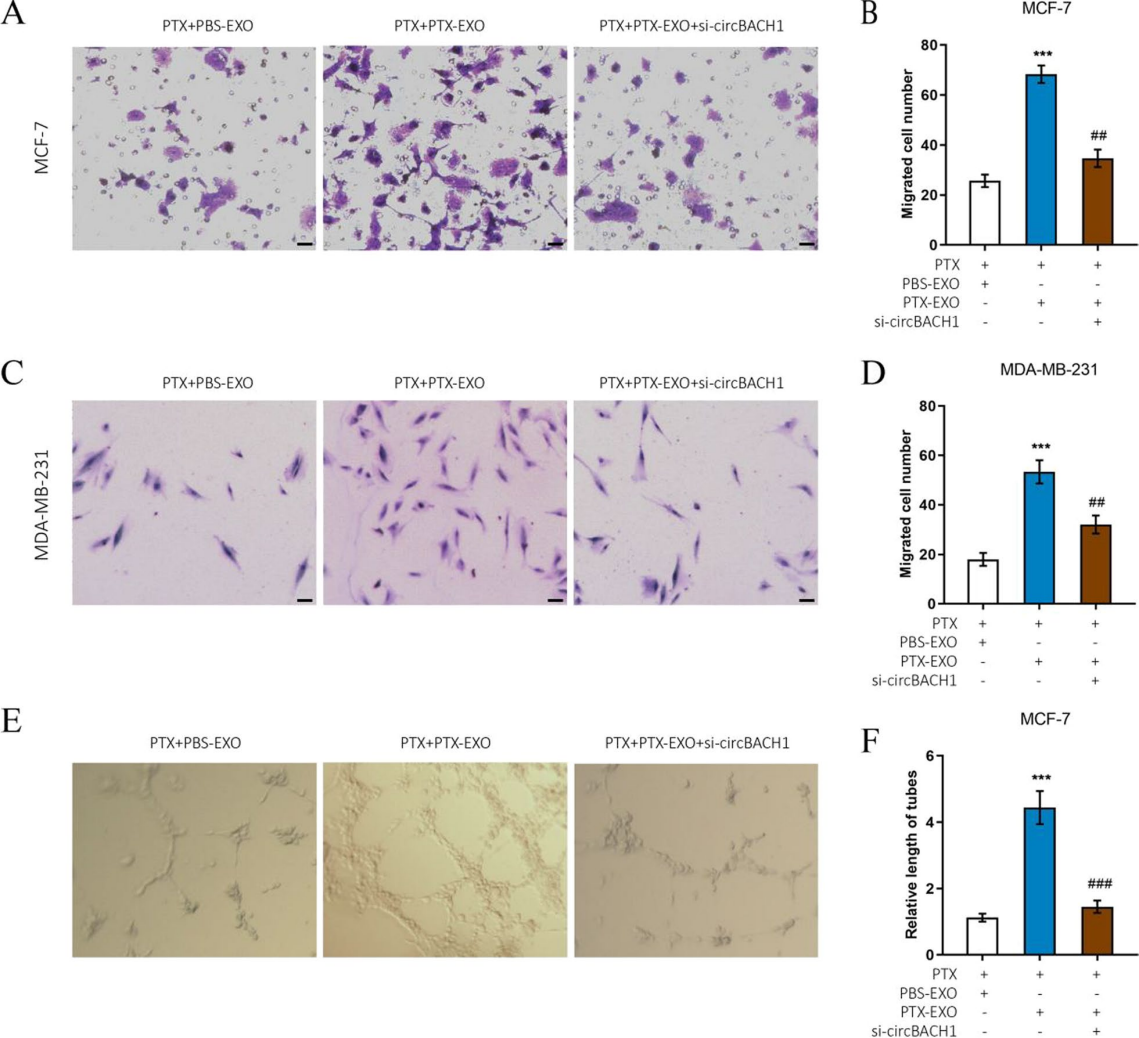
Downregulation of circBACH1 inhibited the PTX-EXO-induced tubes formation of HUVECs. **A**, **C** The MCF-7 and MDA-MB-231 cell migration was detected after treated with PTX and PBS-EXO or PTX-EXO and si-circBACH1, scale bar: 25 µm. The migrated cell number of MCF-7 (**B**) and MDA-MB-231 (**D**) was calculated after treated with PTX and PBS-EXO or PTX-EXO, or si-circBACH1. **E** Representative micrographs of HUVECs forming the tube structures in vitro after 24 h. **F** Quantitative analysis of the tube lengths of HUVECs following treatment with MCF-7 medium after treated with PTX and PBS-EXO or PTX-EXO, or and si-circBACH1. ***P < 0.001, compared with the PTX + PBS-EXO group. *##P* < 0.01, *###P* < 0.001, compared with the PTX + PTX-EXO group

To further confirm that the PTX-EXO mediated the PTX-resistance in the BC cells, the migration was analyzed in MCF-7 and MDA-MB-231 cells. The migrated cell number was significantly increased in the PTX + PTX-EXO group compared with the PTX + PBS-EXO group in MCF-7, MDA-MB-231, and MDA-MB-468 cells (Fig. 4A–D, Additional file 1: Fig. S2A and B). However, the migrated cell number was significantly decreased in the PTX + PTX-EXO + si-circBACH1 group compared with PTX + PTX-EXO group in MCF-7, MDA-MB-231, and MDA-MB-468 cells (Fig. 4A–D, Additional file 1: Fig. S2A and B). To determine whether PTX-EXO affected the angiogenesis of HUVECs, we monitored the tube formation of HUVECs following stimulation with MCF-7 cell medium post corresponding treatment. Compared with the control group, the relative length of tubes in the PTX + PTX-EXO group was substantially increased (Fig. 4E and F). However, the relative length of tubes was significantly decreased in the PTX + PTX-EXO + si-circBACH1 group in comparison to the PTX + PTX-EXO group (Fig. 4E and F). The results demonstrated that PTX-EXO promoted the migration of BC cells and the tube formation of HUVECs, whereas the downregulation of circBACH1 reduced migration to improve PTX-sensitiveness in BC cells, as well as inhibited the PTX-EXO-induced angiogenesis of HUVECs.

### MiR-217 was interacted with circBACH1 in BC cells

**Fig. 5:**
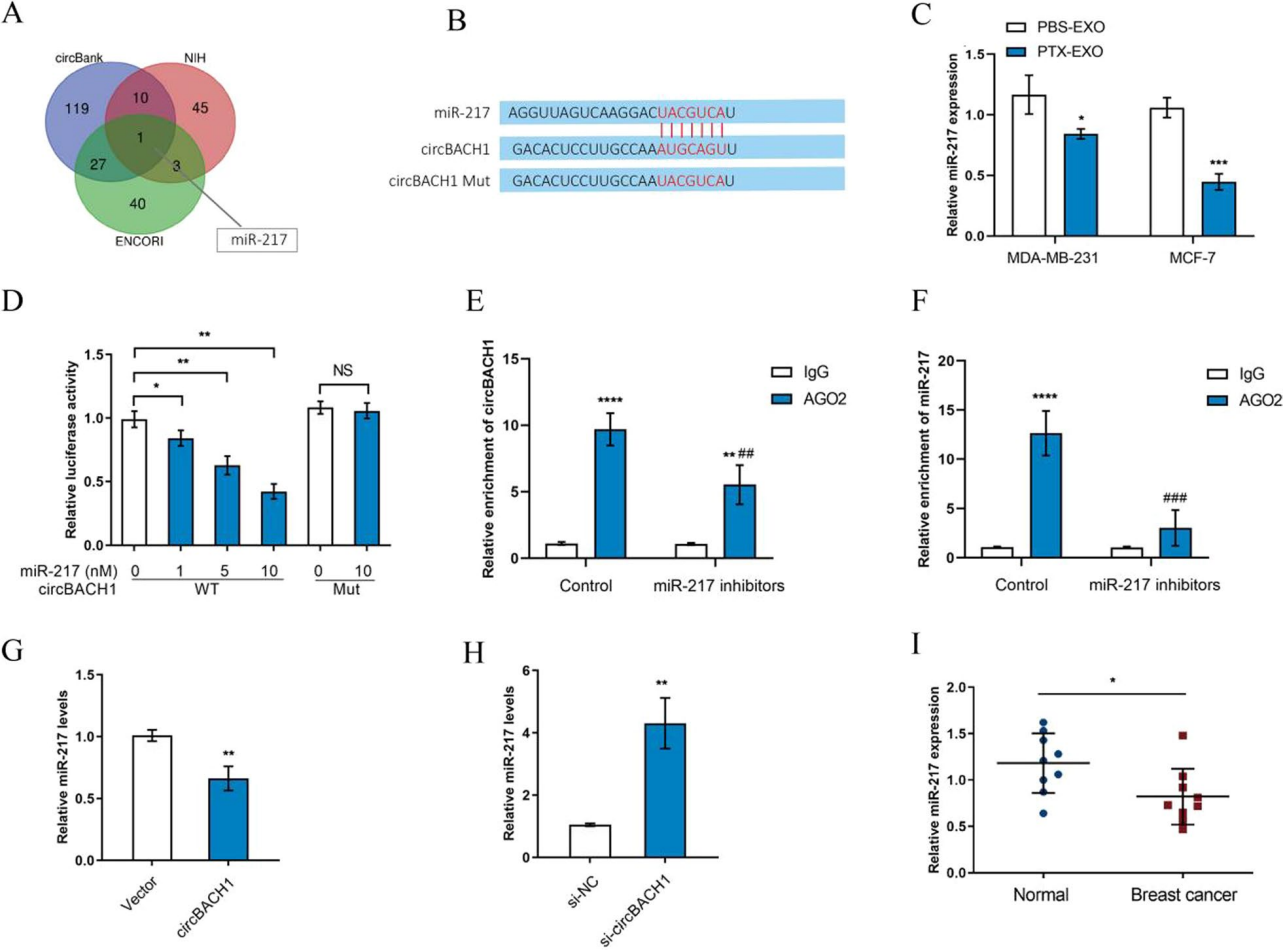
miR-217 was interacted with circBACH1 in BC cells. **A** The Venn diagram of miRNAs predicted to interact with circBACH1 from three database such as circbank, NIH, ENCORI was drawn. **B** The predicted sites of miR-217 and circBACH1 interaction and the mutated sites of circBACH1. **C** The expression of miR-217 was determined by RT-PCR in MCF-7 and MDA-MB-231 cells after PBS-EXO or PTX-EXO treatment. **D** Determining luciferase activity in MCF-7 cells after co-transfected with miR-217 and vectors with wild type and mutant circBACH1 through dual luciferase assay. **E**, **F** The amount of circBACH1 and miR-217 were pulled down by Ago2-RIP assay in MCF-7 cells after treated with miR-217 inhibitors or control. CircBACH1 and miR-217 expression levels were measured by RT-PCR. **G**, **H** The expression of miR-217 in MCF-7 cells was examined by RT-PCR after transfected with vectors or circBACH1 containing vectors (**G**), si-NC or si-circBACH1 (**H**), vetors or si-NC transfection was set as control. **I** miR-217 expression was measured in the tissue of BC patients by RT-PCR, using normal tissue as control. *P < 0.05, **P < 0.01, ***P < 0.001, ****P < 0.0001. *##P* < 0.01, *###P* < 0.001, compared with the control group

To further explore the downstream of circBACH1 regulating PTX-EXO-induced resistance, the interacted-miRNAs were predicted based on the target prediction program of circBank, NIH, and ENCORI. As a result, the overlapped miRNA (miR-217) was obtained from the Venn diagram of the three databases (Fig. 5A). The predicted target sites between circBACH1 and miR-217 were shown in Fig. 5B. To clarify the interaction between miR-217 and circBACH1, the vectors containing wild type or mutant circBACH1 were co-transfected with miR-217 into MCF-7. The luciferase activity diminished gradually with the increased-concentration of miR-217 mimics following co-transfected with the wild type circBACH1, whereas the luciferase activity barely changed with the miR-217 and the mutant circBACH1 co-transfection (Fig. 5C). RNA-IP results showed that the relative abundance of circBACH1 and miR-217 in the anti-Ago2 group significantly increased compared to the anti-IgG group, whereas in the miR-217 inhibitors group, the relative abundance of circBACH1 and miR-217 immunoprecipitated with Ago2 was lower than that in the control group (Fig. 5D and E). To further validate the interaction between circBACH1 and miR-217, miR-217 expression levels were detected following PTX-EXO treatment in BC cells. The results showed that the expression levels of miR-217 were significantly decreased in PTX-EXO-treated MDA-MB-231 and MCF-7 cells compared with the control (Fig. 5F). Consistently, the expression levels of miR-217 were also significantly reduced in PTX-treated BC tissue in comparison to the adjacent tissue (Fig. 5G). Moreover, the expression levels of miR-217 reduced significantly following the overexpression of circBACH1 compared with control, whereas miR-217 expression was obviously increased following transfection with si-circBACH1 compared with control in MCF-7 cells (Fig. 5H and I). In addition, we also found that the mammosphere formation was significantly reduced in miR-217 mimics-treated MCF-7 cells compared with control (Additional file 1: Fig. S2C and D).

### Downregulation of G3BP2 suppressed BC cell migration

**Fig. 6:**
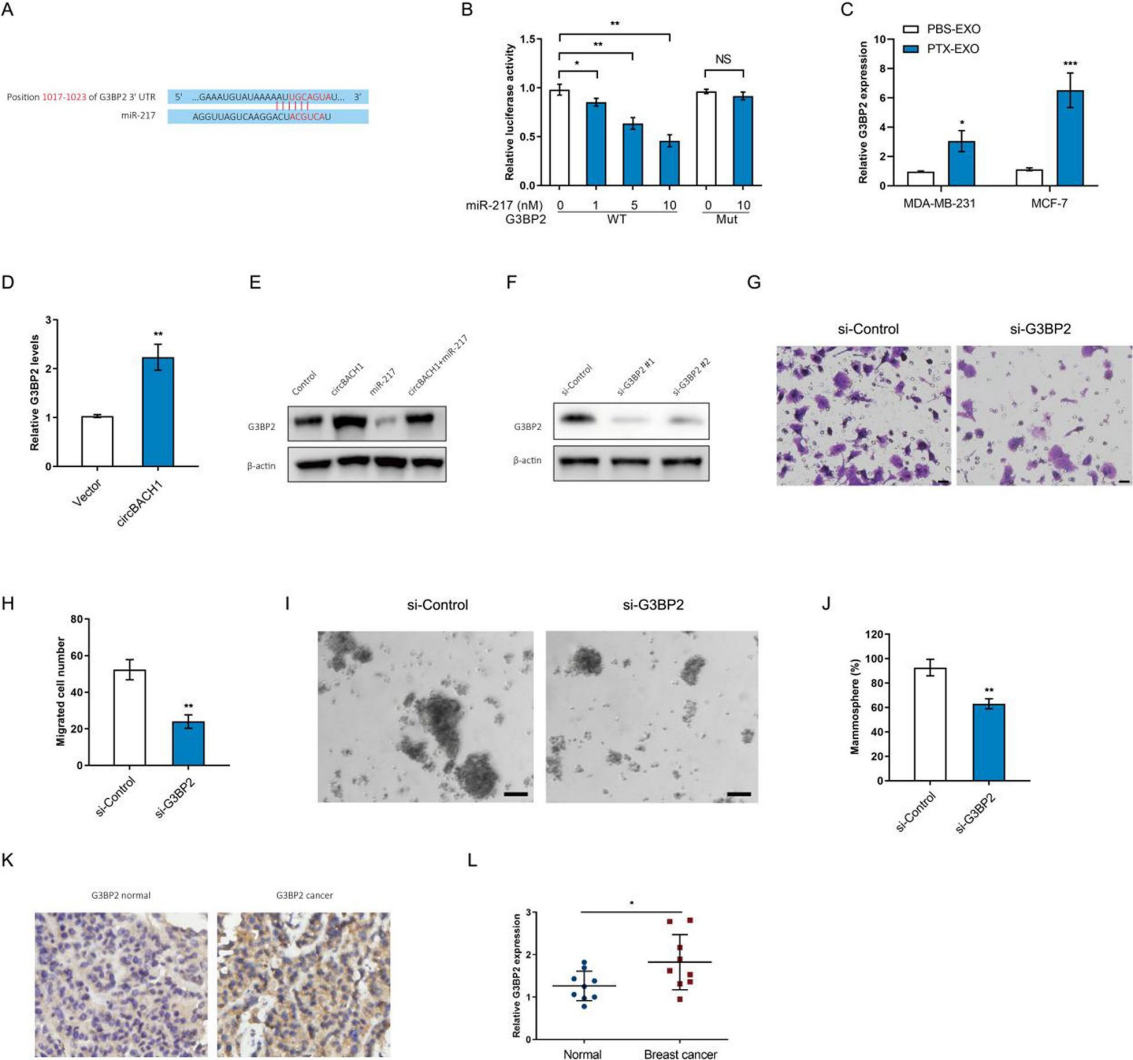
Downregulation of G3BP2 suppressed BC cell migration and stemness. **A** The predicted sites of target gene G3BP2 and miR-217 interaction. **B** Determining luciferase activity after co-transfected miR-217 and vectors with wild type and mutant G3BP2 into MCF-7 cells through dual luciferase assay. **C** The expression levels of G3BP2 were determined in MCF-7 and MDA-MB-231 cells after PBS-EXO or PTX-EXO treatment. **D** The G3BP2 levels were detected by RT-PCR in MCF-7 cells transfected with vectors or circBACH1 containing vectors, vetors transfection was set as control. **E** Western blot assay was performed to determine the G3BP2 proteins levels in MCF-7 cells transfected with circBACH1 or miR-217 vectors. **F** The G3BP2 protein levels were measured by western blotting assay after downregulation of G3BP2 mRNA. **G**, **H** Determination of the migrated cell number of MCF-7 cells transfected with si-G3BP2 by Transwell assay, scale bar: 25 µm. **I**, **J** Mammosphere formation (%) was determined in MCF-7 cells treated with si-G3BP2, scale bar: 50 µm. **K** The G3BP2 expression was detected by immunohistochemistry in tissue of BC patients, using normal tissue as control (×400). **L** The G3BP2 expression was measured in tissue of BC patients by RT-PCR, using normal tissue as control. *P < 0.05, **P < 0.01, ***P < 0.001

miR-217 was involved in the suppression of the proliferation, invasion, and migration of cancer cells [32–34]. To explore the downstream mechanism of miR-217, the target gene was predicted by TargetScan 7.2 program. G3BP2 was an oncogene involved in breast cancer progression and metastasis [35]. The predicted binding sites of miR-217 in G3BP2 mRNA 3'UTR were provided by the TargetScan 7.2 program (Fig. 6A). The luciferase reporter vectors containing wild type or mutant G3BP2 and miR-217 were co-transfected into MCF-7 cells. Interestingly, the luciferase activity of wild type G3BP2 was significantly reduced in the increased concentration of miR-217 compared with control, whereas the luciferase activity of mutant G3BP2 barely changed between miR-217 group and control group in MCF-7 cells (Fig. 6B). We observed that the expression levels of G3BP2 were significantly increased in PTX-EXO-treated MDA-MB-231 and MCF-7 cells compared with the control (Fig. 6C). Moreover, overexpression of circBACH1 increased the G3BP2 levels in MCF-7 cells compared with control (Fig. 6D). We determined the expression of the G3BP2 proteins in MCF-7 cells by western blot assay after cotransfected with circBACH1 and miR-217. The G3BP2 proteins were upregulated by circBACH1 transfection, while downregulated by miR-217 transfection compared with control in MCF-7 cells (Fig. 6E). However, circBACH1 and miR-217 cotransfection suppressed the expression of G3BP2 proteins compared with circBACH1 treatment in MCF-7 cells (Fig. 6E). To elucidate the effect of G3BP2 on BC progression, G3BP2 was downregulated in MCF-7 cells. The protein expression of G3BP2 was testified following downregulation of G3BP2 (Fig. 6F). To explore the effect of G3BP2 on BC cell function, cell migration and mammosphere formation were determined following downregulation of G3BP2. The migrated cell numbers were significantly reduced by treatment with si-G3BP2 #1 compared with control in MCF-7 cells (Fig. 6G and H). Downregulation of G3BP2 significantly attenuated mammosphere formation compared with control in MCF-7 cells (Fig. 6L and J). Consistent with in vitro results, the G3BP2 levels were increased in BC tissue compared with the adjacent tissue, which was confirmed by IHC assay and RT-PCR (Fig. 6K and L).

## Discussion

Exosomes consist of an information transmission system among cells to transfer materials and information, which play important roles in cancers including tumor microenvironment remodeling, angiogenesis, metastasis, and drug-resistance [36]. Exosome-carried signals regulate the complex intracellular pathway for adjacent cells or distant cells to create an appropriate condition for BC initiation and aggressiveness [37]. BC derived exosomes influence the transcription and related signaling pathways of target cells to cause angiogenesis, therapeutic resistance, and metastasis [38, 39]. Extensive studies have shown that BC derived exosomal non-coding RNAs, proteins, and DNAs were diffferently expressed, which were viewed as potential indicators for diagnosis or prognosis in BC [39–42]. A previous study has demonstrated that BC derived exosomes enhanced BC aggressiveness by transferring miR-370-3p to fibroblasts and activating the fibroblasts through NF-κB pathway [43]. Our study demonstrated that PTX-EXO promoted cell viability, stemness, and metastasis of MDA-MB-231 and MCF-7 during PTX treatment, suggesting that PTX-EXO might be involved in BC progression. The increased cell viability, stemness, metastasis in BC cells indicated that the PTX-sensitivity was reduced by PTX-EXO, which indicated that exosomes exerted an important role in PTX-resistance of BC cells.

The mechanism of PTX-resistance is a complex, and involves various aspects including drug efflux, energy metabolism, cancer cell mutation, and autophagy [44–46]. Previous studies reported that BC cells derived exosomes promoted multiple drugs resistance including doxorubicin, docetaxel, cisplatin, and tamoxifen resistance via BC miRNAs [47–49]. A study showed that miR-378a-3p and miR-378d were increased in BC cells derived exosomes, which promoted BC stemness and chemoresistance [50]. Upregulated circ-RNF111 has been demonstrated to inhibit miR-140-5p expression in PTX-resistant BC tissues and cells to promote cell viability, cell invasion of BC [45]. Another latest study demonstrated that exosomal circHIPK3 derived from BC cells enhanced the angiogenesis and the tube formation of human endothelial cells by upregulating MTDH through miR-124-3p [38]. The underlying molecular mechanism of PTX-resistance in BC remains to be elucidated. CircRNA is a special circular single-strand RNA produced by precursor mRNA back-splicing, which plays roles in serving as miRNAs sponges, RNA binding proteins sponges, proteins/peptide translators, and transcription regulators [51–56]. In this study, we found that the expression of circBACH1 was significantly increased in PTX-EXO of MCF-7 and MDA-MB-231. The inhibition of circBACH1 significantly reversed the PTX-EXO enhanced cell viability, migration, stemness of BC cells, and the PTX-EXO enhanced angiogenesis of HUVECs, which suggested that circBACH1 was a potential therapeutic target for chemoresistance and stemness in BC.

**Fig. 7:**
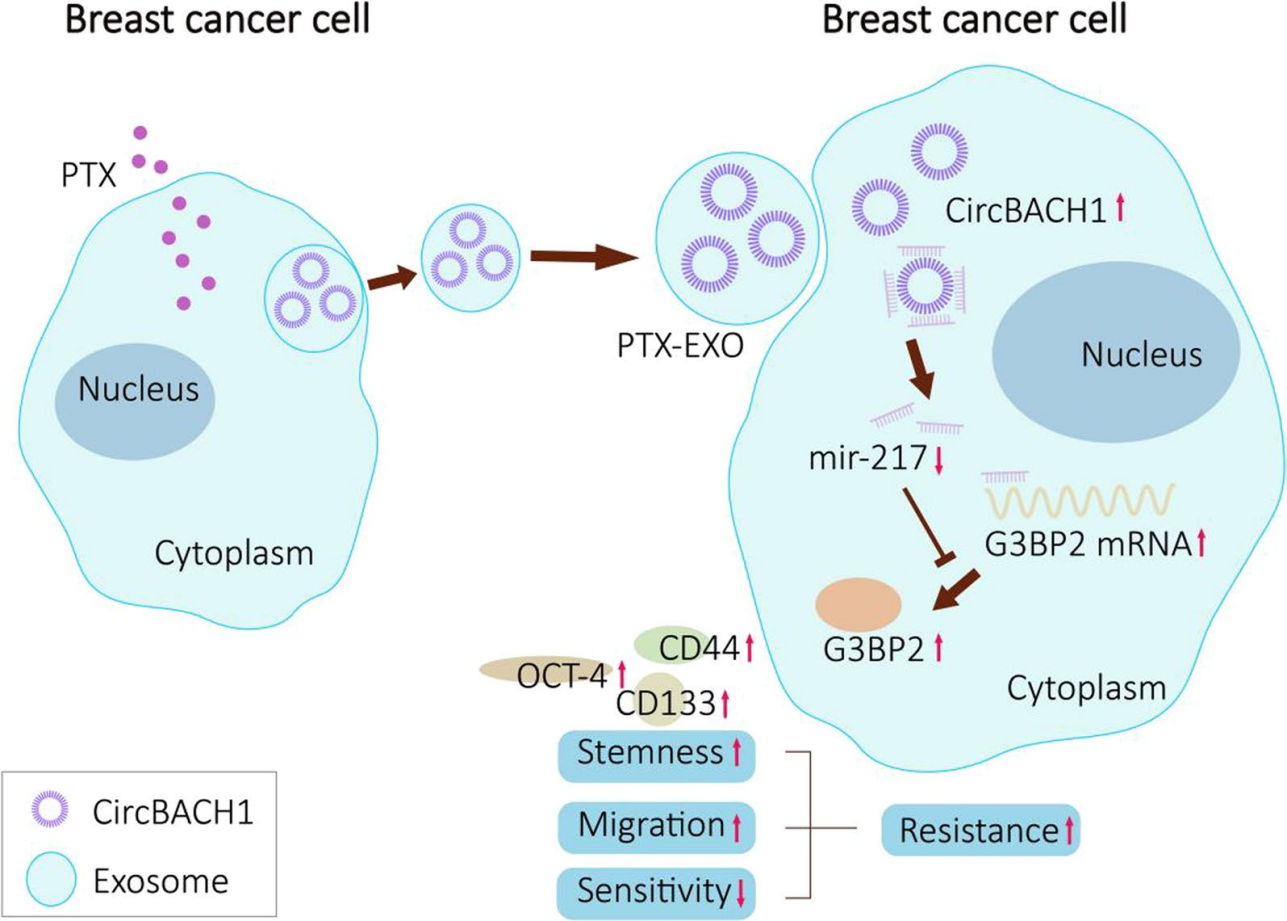
The schematic diagram of PTX-EXO-induced circBACH1/miR-217/G3BP2 axis regulating breast cancer cell resistence. PTX-EXO accelerates BC cell resistence via activating circBACH1/miR-217/G3BP2 signaling pathway. CircBACH1 promoted stemness and migration of BC cells by sponging miR-217 to up-regulate the expression of G3BP2

MiRNAs are small non-coding RNAs around 20–24 bps, which involve in cell cycle including cell proliferation, differentiation, and death [54]. MiRNAs regulate transcription, translation, and degradation of mRNAs to mediate target genes [55]. Massive miRNAs have been reported to be promising biomarkers for BC metastasis such as lymph node metastasis, bone metastasis, brain metastasis, and distant metastasis [39, 40, 54]. Previous studies showed that miR-217 inhibited the proliferation, invasion, and migration of several cancer cells including prostate cancer cells, pancreatic cancer cells, and pulmonary carcinoma cells, due to the suppression of target gene expression and relative signaling pathway [32–34]. Besides, a recent study demonstrated that miR-217 was strongly down-regulated in pancreatic cancer, whereas miR-217 mimic transfection enhanced pancreatic cancer cells sensitivity to gemcitabine mainly through the suppression of several drug resistance genes [56]. Additionally, it has been verified that G3BP2, an oncogene for BC was negatively regulated by miRNA to inhibit osteosarcoma and bladder cancer progression [30, 57]. Our study verified that the expression of miR-217 was reduced, while G3BP2 was overexpressed in BC tissues. G3BP2 was validated as the direct target of miR-217 by luciferase assay. Inhibition of G3BP2 expression suppressed the cell migration of BC cells. Together, these results demonstrated that PTX-EXO promoted BC cell resistance via activating circBACH1/miR-217/G3BP2 signaling pathway (Fig. 7).

BC is a highly heterogeneous neoplasm, which is mainly classified into luminal, basal, and HER2 subtypes. Basal subtype BC negatively expressed estrogen receptor (ER), progesterone receptor (PR), and HER2, while luminal A subtype BC was ER^+^ or PR^+^, and HER2^−^. The purpose of this study is to clarify the mechanism of chemoresistance and metastasis of luminal A and basal subtype BC. The low-aggressive luminal A subtype BC cell MCF-7, basal subtype BC cell MDA-MB-468, and the high-aggressive basal subtype BC cell MDA-MB-231 were used as the in vitro models to explore the mechanism of PTX-resistance and progression of BC. The expression of circBACH1 was increased post PTX treatment in BC cells derived exosomes and the lysates of MDA-MB-231 cells. Whereas no significant changes in the circBACH1 expression were observed post PTX treatment in the lysates of MCF-7 cells. However, the circBACH1 expression was substantially increased post PTX treatment in the exosomes of MCF-7 cells, and we validated that circBACH1 was mainly located in cytoplasm of MCF-7 cells. The results implied that circBACH1 was probably more stable in exosomes of MCF-7 cells thus leading to the more circBACH1 excluded by exosomes. Intriguingly, PTX-EXO treatment led to an increase of circBACH1 expression in the lysates of MCF-7 cells in comparison to MDA-MB-231 cells, suggested that MCF-7 cells might be more sensitive to PTX-EXO. PTX-EXO treatment induced PTX-resistance through transferring circBACH1. A latest study indicated that endocrine resistance was transcriptomic regulated by differentially expressed lncRNA, circRNA, microRNA and mRNA in endocrine resistant breast cancer cells compared with endocrine sensitive MCF-7 [58]. It has been demonstrated that circRNAs act as miRNA sponge to regulate endocrine resistance [24, 59]. In our study, we demonstrated that PTX-induced exosomal circBACH1 regulated stemness and migration of BC cells by sponging miR-217 to upregulate the expression of G3BP2, which provided a new therapeutic target for PTX-resistance and progression of BC via circBACH1/miR-217/G3BP2 axis.

## Conclusion

In summary, we clarified that the exosomal circBACH1 was increased in BC post PTX-therapy in vitro and clinically, which was the leading factor to the obtained PTX-resistance for BC. Furthermore, we showed that the suppression of circBACH1 effectively inhibited cell viability, migration, stemness in MCF-7 and MDA-MB-231, and angiogenesis in HUVECs. Moreover, we clarified that the effect of circBACH1 on BC progression was attributed to sponging miR-217 to upregulate G3BP2 expression. The results indicated that circBACH1/ miR-217/G3BP2 axis was a new regulatory strategy for PTX-resistance and progression of BC.

## Supplementary Information

**Additional file 1**. Downregulation of circBACH1 reversed PTX-EXO-induced cell migration.

## Data Availability

The datasets used or analyzed during the current study are available from the corresponding author on reasonable request.
