# Supplementary material for: Chemotherapy-induced exosomal circBACH1 promotes breast cancer resistance and stemness via miR-217/G3BP2 signaling pathway
Source: Breast Cancer Res. 2023 Jul 17;25:85. doi: 10.1186/s13058-023-01672-x (PMC10351125; doi:10.1186/s13058-023-01672-x)
Supplement: Supplementary file 1 — Additional file 1. Downregulation of circBACH1 reversed PTX-EXO-induced cell migration. [file 13058_2023_1672_MOESM1_ESM.docx]

**Supplementary figures and figure legends**


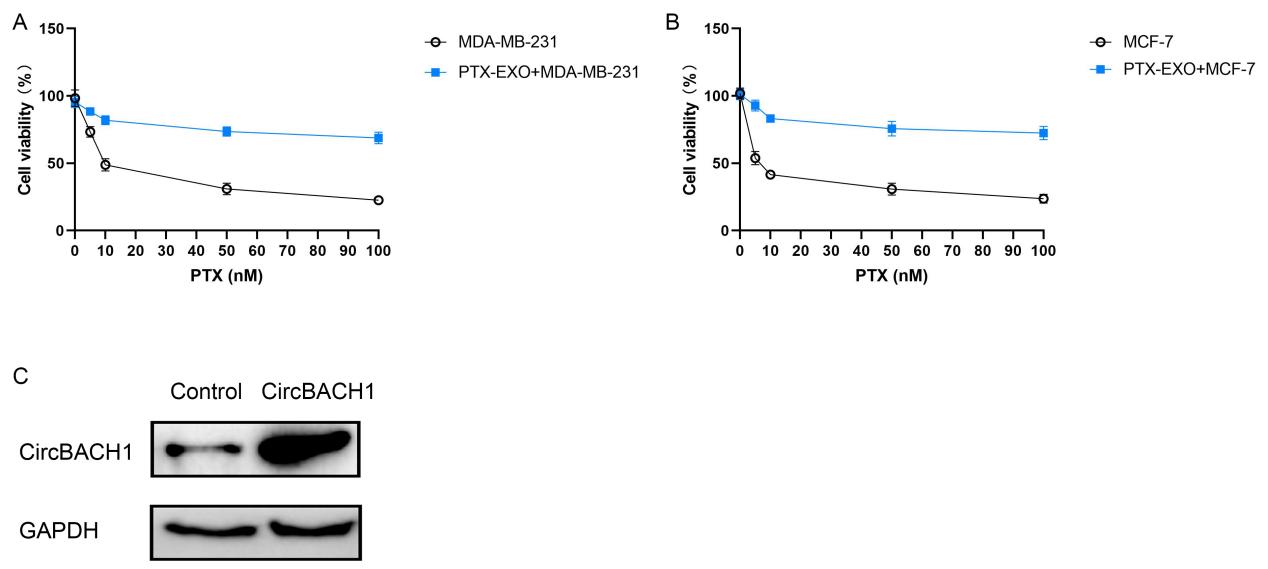


**Supplementary Figure 1. PTX induced cytotoxicity in MDA-MB-231 and MCF-7 cells, but not in PTX-EXO treated in MDA-MB-231 and MCF-7 cells.** Cytotoxicity of MDA-MB-231 (A) and MCF-7 (B) cells induced by different concentration of PTX. (C) Northern blotting of circBACH1 expression by transfection with circBACH1 vectors.


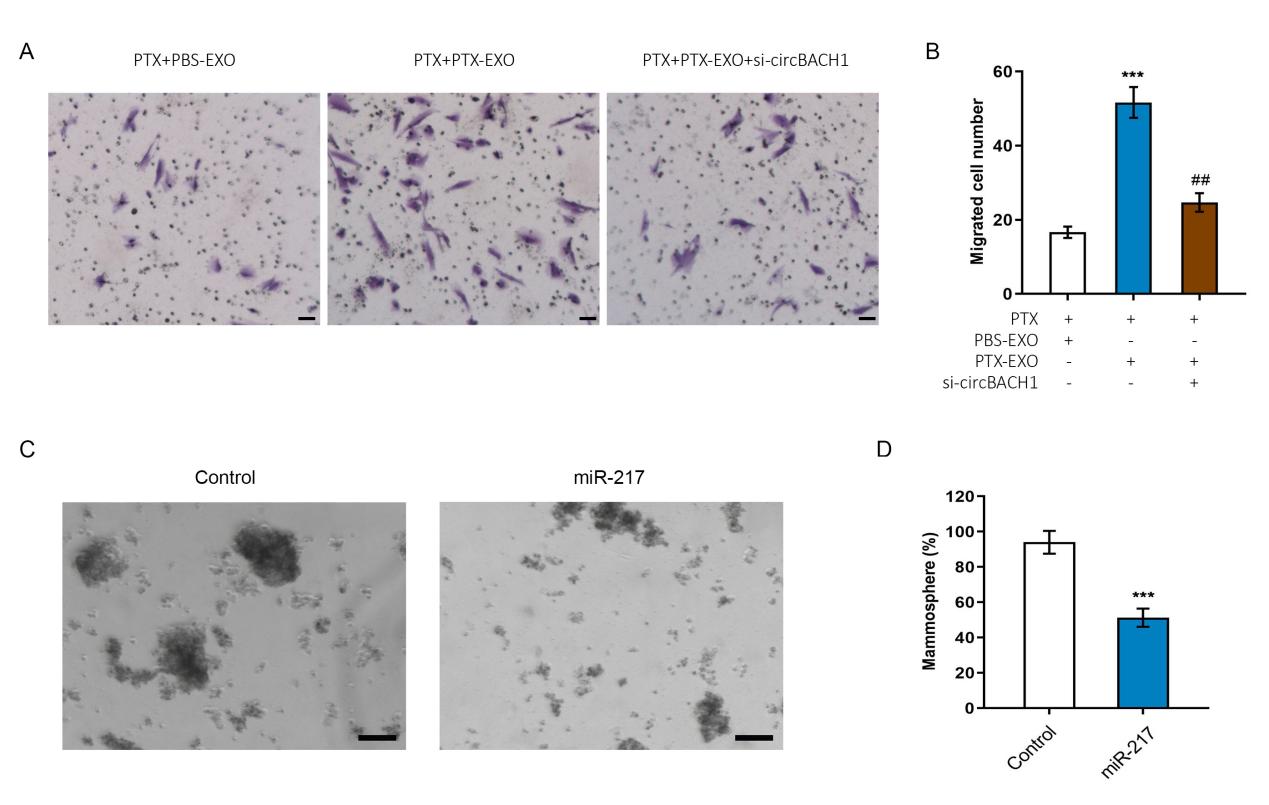


**Supplementary Figure 2. Downregulation of circBACH1 reversed PTX-EXO-induced cell migration.** (A, C) The cell migration was determined in MDA-MB-468 cells after treated with PTX and PBS-EXO or PTX-EXO and si-circBACH1, scale bar: 25 µm. (B) The migrated cell number of was calculated in MDA-MB-468 cells after treated with PTX and PBS-EXO or PTX-EXO, or si-circBACH1. *** *P* < 0.001, compared with the PTX + PBS-EXO group. *## P* < 0.01, compared with the PTX + PTX-EXO group. (C, D) Mammosphere formation (%) was determined in MCF-7 cells treated with miR-217 mimics, scale bar: 50 µm. *** *P* < 0.001.
